# Reinforcement Learning with Side Information for the Uncertainties

**DOI:** 10.3390/s22249811

**Published:** 2022-12-14

**Authors:** Janghoon Yang

**Affiliations:** Department of A.I. Software Engineering, Seoul Media Institute of Technology, Seoul 07590, Republic of Korea; jhyang@smit.ac.kr; Tel.: +82-2-6393-3237

**Keywords:** reinforcement learning, consensus, multi-agent system, sliding mode control

## Abstract

Recently, there has been a growing interest in the consensus of a multi-agent system (MAS) with advances in artificial intelligence and distributed computing. Sliding mode control (SMC) is a well-known method that provides robust control in the presence of uncertainties. While our previous study introduced SMC to the reinforcement learning (RL) based on approximate dynamic programming in the context of optimal control, SMC is introduced to a conventional RL framework in this work. As a specific realization, the modified twin delayed deep deterministic policy gradient (DDPG) for consensus was exploited to develop sliding mode RL. Numerical experiments show that the sliding mode RL outperforms existing state-of-the-art RL methods and model-based methods in terms of the mean square error (MSE) performance.

## 1. Introduction

With the evolution of network technologies, previously standalone devices were getting connected to the network, thereby achieving the vision of the “internet of everything”. Accordingly, the network plays a critical role in a control system, often called a networked controlled system. Many networked controlled systems comprise multiple subsystems which can be modules for specific functions or agents to operate independently. Although decentralized control has been developed as an alternative to a centralized control to overcome the complexity issue, its performance can be degraded owing to limited information. Distributed control is known to provide a tradeoff between complexity and performance by utilizing information from a subset of agents in the system.

The objective of the distributed control usually depends on the goal of the system. A computational model for the distributed control of a system with sensing and actuation operating over wireless sensor networks was proposed to address issues intrinsic to the sensor network, such as communication jitter [1]. A distributed proportional-integral-derivative (PID) controller was derived by formulating primal-dual dynamics to maximize the sum network utility maximization with a link capacity constraint [2]. A model predictive distributed control for a system consisting of interacting subsystems was developed in the context of min-max optimization to derive robust distributed control [3]. Its significance has been shown to increase in association with cyber-physical systems (CPS) such as the cooperative control of manipulators and multi-agent system (MAS) such as microgrids [4]. The MAS is usually defined as a system comprising a set of agents for performing a task [5]. One of the most popular problems associated with it is the consensus problem in which each agent executes an action to achieve a common goal. Consensus control can be considered as a type of distributed control in which each agent shares its state information with neighbor agents, from which a consensus control is determined. Consensus control for the MAS has been studied in many practical problems such as a swarm of unmanned aerial vehicles (UAVs) [6], autonomous vehicle platoon [7], reactive power control in microgrids [8], and teleoperation of cyber-physical systems [9].

For simplicity of analysis, some studies assume that there is no delay associated with exchanging information. However, communication delays are unavoidable in practical systems, and various approaches have been proposed to address them. When the delays are the same for all communication links, the conventional consensus protocol achieves consensus as long as the delay is less than the threshold determined by the algebraic connectivity of the communication graph [10]. Similarly, an asynchronous event-triggered consensus protocol for MAS with second-order dynamics and the same delay was shown to achieve an average consensus when the delay is less than the event detection period [11]. An observer was introduced to predict the state of the MAS with both delay and disturbance [12] and cooperative containment control with delay [13]. The consensus conditions have been given a linear matrix inequality (LMI) from the Lyapunov stability condition for a homogenous MAS with a Markov delay [14], heterogeneous MAS with random link failures [15], and MAS with higher-order dynamics and multiple time-varying delays [16]. A bounded delay condition for the consensus of a heterogeneous MAS has also been developed using a frequency domain method [17].

Because delayed information is different from information without delay, it can be considered uncertain. A popular method for developing robust control involves exploiting sliding mode control (SMC). It is a nonlinear control in which the switching operation induces the state of the system into the sliding surface which can be defined independently from the original system dynamics and uncertainties [18]. SMC has been introduced to address different types of uncertainties and system configurations associated with the consensus of MAS such as an affine nonlinear MAS with disturbances and system uncertainties [19], heterogeneous second-order MAS with uncertain parameters [20], nonlinear MAS with communication delay [21], second-order MAS with constant same input delay and disturbance [22], and second-order MAS with un-known time-varying delays and disturbances [23]. Alternatively, reinforcement learning (RL) can be introduced to develop robust consensus control. RL learns to generate an action to maximize the expected return. Most existing RL-based consensus controls were developed from adaptive dynamic programming by solving coupled Hamilton–Jacobian–Bellman (HJB) equations [24,25,26]. They focused on the development of a consensus algorithm without explicit model knowledge, rather than dealing with uncertainties. In addition, most existing RL algorithms are sensitive to parameterizations and convergence problems [24].

When multiple agents move together, their communication links become fragile. Thus, centralized control may not be feasible, necessitating distributed consensus control. In addition, there are uncertainties in the system model owing to uncertainties such as disturbances and delays. The recent success of RL in many control problems and the robust performance of SMC in the presence of uncertainties motivated this study. Many existing consensus control algorithms depend on model knowledge to derive a control signal from an assumed model. However, accurate system modeling is often limited, which results in degraded control performance. Alternatively, an RL approach that utilizes learning through experience without specific model knowledge may be adopted. However, most existing RL algorithms are sensitive to parameterizations and convergence problems [27].

Our previous work showed that, although the application ofa twin-delayed deep deterministic policy gradient (DDPG) with articulated reward shaping provides a robust performance of the consensus, its performance is limited in comparison to that of the model-based algorithm [28]. Ref. [29] combined SMC with RL for consensus control in the presence of uncertainties, which was called the “slide RL”. The performance of the combined method is comparable to that of model-based control. However, slide RL works only when the parameters are initialized with values close to zero, and the update rate is very small. Thus, it is very sensitive to parameterizations. Because the slide RL is based on the RL developed from the coupled HJB equation, its development for various types of control problems necessitates mathematical development for approximate dynamic programming. This can be very difficult to derive for a system with particular dynamics. Thus, to develop a consensus algorithm whose performance is robust to uncertainties and parameterization, and comparable to that of the model-based algorithm, we combine SMC with the state-of-the-art RL and twin delayed DDPG called TD3 [30]. This is called the “sliding mode RL” to distinguish it from the slide RL. The sliding mode RL is designed such that the switching control provides robustness to uncertainties, while the RL-based control improves the performance further by exploiting the SMC structure. The simulation result shows that the sliding mode RL achieved the best mean square error (MSE) performance in comparison to the model-based consensus algorithm and other RL-based algorithms. To further analyze the characteristics of the sliding mode RL, the performance of the switching control (SC), which is an SMC without a linear control part, was assessed. Interestingly, the performance of the SMC without linear control was comparable to that of other methods considered. This result suggests that sliding mode RL benefited considerably from nonlinear sliding control rather than RL. Transmission over wireless communication links incurs delays owing to distance, processing delays, and channel errors. In addition, disturbances, such as wind and rain, can occur in the moving path, and it is often difficult to obtain a precise mathematical model to derive a control signal. Thus, it is important to develop a robust control method without explicit knowledge of the system model. The remainder of this study is organized as follows: a system model for a multi-agent system and the objective of the consensus of MAS in mathematical form are presented in Section 2. The proposed sliding mode RL and its pseudo-code are described in Section 3. The numerical results which verify the performance of the sliding model RL are presented in Section 4. Section 5 contains concluding remarks and directions for future research.

## 2. System Model and Formulation of the Problem

**Figure 1:**
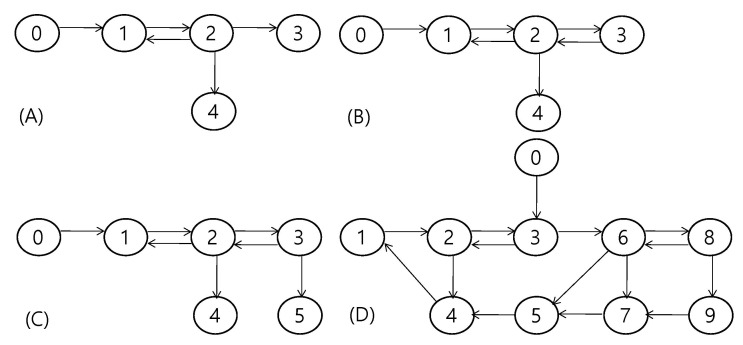
(**A**–**D**) Communication graphs for simulations.

In this study, we considered a second-order homogeneous MAS with a single leader and multiple followers in an environment with unknown time-varying delays and disturbances. Figure 1 depicts the four different realizations of the communication graphs, where node 0 is considered the leader node for all graphs. Each agent is assumed to transmit information to neighboring nodes that are connected through an edge in a communication graph. A leader–follower MAS aims to achieve a consensus on the position of the leader agent by sharing information with neighbors connected through communication links. The corresponding behavior of each agent can be expressed as

(1) $${\ddot{x}}_{i}(t)=u_{i}(t)+d_{i}(t),$$

where $u_{i}(t)$, $d_{i}(t)$, $x_{i}(t)$, and ${\ddot{x}}_{i}(t)$ denote the control signal of agent *i*, an unknown bounded disturbance, the position of agent *i*, and second-order derivative of $x_{i}(t)$, respectively. Since $u_{i}(t)$ is determined from the information available at agent *i*, it can be expressed as

(2) $$u_{i}(t)=f(H(X_{i}(t),X_{i^{-}}(t))),$$

where H(⋅) is the history of inputs from the initial time to time *t*, $X_{i}(t)=[x_{i}(t),\;{\dot{x}}_{i}(t)]$, $X_{i^{-}}(t)=\cup_{j\in N_{i}}\left\{x_{j}(t-\tau_{i,j}(t)),\;{\dot{x}}_{j}(t-\tau_{i,j}(t))\right\}$, $N_{i}$ is a set of neighbor agent indices of agent *i*, $\tau_{i,j}(t)$ is a communication delay at time *t* from agent *j* to agent *i*, and f(⋅) is a mapping whose output is an action.

The objective of the control of a MAS considered in this research is to achieve positional consensus to the leader agent, which can be expressed as

(3) $$\lim_{t\to \infty }|e_{d,}{}_{i}(t)|=0\;\;for\;\;\forall i,$$

where $e_{d,}{}_{i}(t)=x_{i}(t)-x_{0}(t)$. $e_{d,}{}_{i}(t)$ is often referred to as a disagreement vector. To achieve (3), consensus control is performed independently for each agent using the available information. The convergence of the consensus of MAS with the knowledge of a perfect system model and an introductory explanation of the consensus are provided in [10]. However, the leader agent may send its state information to neighbor agents only. In addition, there may be a delay in the communication links. Thus, information on the disagreement vector is often unavailable. Alternatively, the local position error $e_{x,}{}_{i}(t)$ and local velocity error $e_{v,}{}_{i}(t)$ are used to generate the control signal:

(4) $$e_{x,i}(t)=\sum_{j\in N_{i}}x_{i}(t)-x_{j}(t-\tau_{i,j}(t)),$$

(5) $$e_{v,i}(t)=\sum_{j\in N_{i}}{\dot{x}}_{i}(t)-{\dot{x}}_{j}(t-\tau_{i,j}(t)),$$

## 3. A Sliding Mode RL for the Consensus of a MAS

In this section, a framework for developing a sliding mode RL is explained first so that it can be applied to most existing RLs independently from the specific algorithm structure in RL. Then, the sliding mode RL based on TD3 is presented with a pseudo-code as a specific realization. The time index is omitted for simplicity.

RL is a model-free algorithm for determining the policy of an agent through interactions with the environment which follows the Markov decision process (MDP). When the model information, which is transition probability, is given, it can be solved using the Bellman equation satisfied by the optimal policy π* [30]:

(6) $$Q^{\pi *}(s,a)=r(s,a)+\gamma E_{s^{\prime }a^{\prime }}\{Q^{\pi *}(s^{\prime }a^{\prime })\},$$

where *a* tuple $\left(s,a,r,s^{\prime },a^{\prime }\right)$ which consists of the current state s, current action a, reward r, next state $s^{\prime }$, and next action $a^{\prime }$ defines an experience used to update the RL. $Q^{\pi }(s,a)$ is called a state–action value function, which is determined by a policy π.

The control signal with SMC can be expressed as

(7) $$u_{SMC}=f(x_{0},\cdots ,x_{K})-k_{u}sign(q),$$

where f(⋅) is a function that is determined from the definition of the sliding variable and dynamicity of the agents, K is the number of follower agents, $k_{u}$ is a nonlinear control gain that determines the degree of robustness to uncertainties, and q is the sliding variable. The proposed method combines RL and SMC such that f(⋅) can be determined through RL. Nonlinear switching control in the second part of Equation (7) is kept the same as in SMC. The corresponding sliding mode RL is expressed as (8)

(8) $$u_{sliding\;mode\;RL}=A_{\phi }(s)-k_{u}sign(q)+\varepsilon ,$$

where s is the state information used for generating an action, $A_{\phi }(\cdot )$ is the output of a neural network parameterized by ϕ, and ε is the exploration noise of which distribution can be defined in an implementation-specific way.

**Figure 2:**
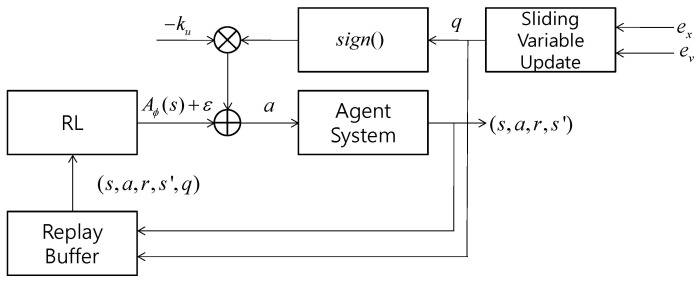
Structure of sliding mode RL.

**Figure 3:**
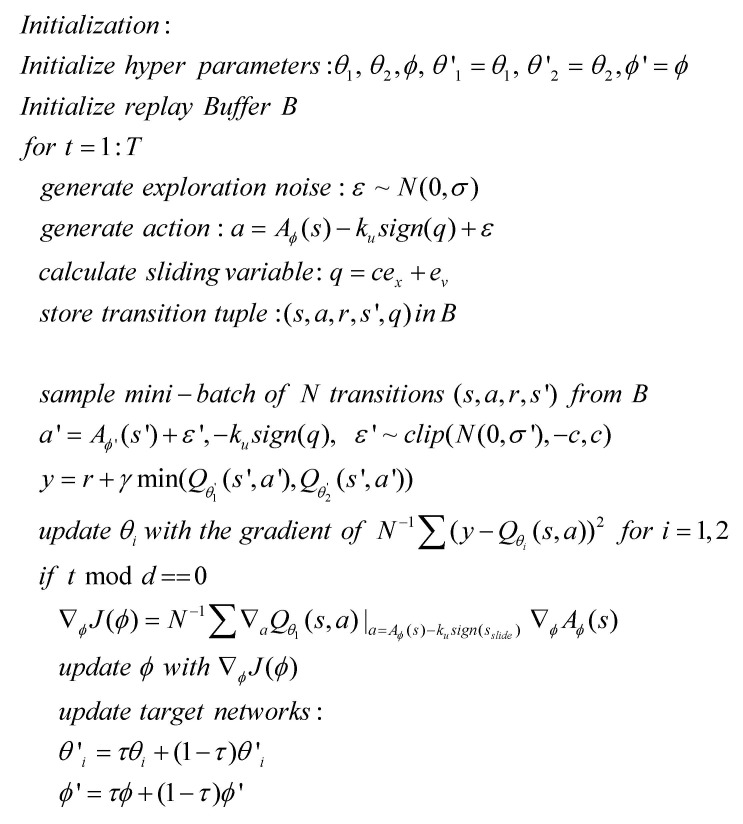
Pseudo-code for the sliding mode TD3.

TD3 [30] is considered as a specific method to be used for developing the proposed algorithm. The structure of the proposed sliding mode RL is shown in Figure 2. RL generates an action for refining the switching control, while the sliding variable is updated with the position and velocity errors at each time step. The control signal a is generated by Equation (8) and applied to the agent. A resulting tuple of experience $\left(s,a,r,s^{\prime },q\right)$ is stored in the replay buffer from which the batch training data are sampled to train the neural networks for RL. TD3 was designed to reduce the overestimation bias by using a pair of independent critics, considering the target action–value function with clipped double Q learning and delaying policy updates. The pseudo-code for the sliding mode TD3 is presented in Figure 3 through modifying the pseudo-code of TD3 in [30]. It starts with initializing hyper-parameters and replaying buffer. At each time step, it generates an action which can be decomposed into SMC and exploration noise. The SMC is further decomposed into the refinement control, which is generated by the actor network, and the switching control, which is driven by local errors. Then, the tuple of the experience at the current time step is saved at the replay buffer. Now, N samples (N is the batch size) are sampled from the replay buffer to train the network for RL. After generating the action for the next state from the actor network and clipped exploration noise, the target Q value is calculated from the double Q values from target critic networks. The critic networks are then updated. If the current time step t is the integer multiple of the target update period, the actor networks are updated by the deterministic policy gradient. The target critic and actor networks are also updated with the parameters of the main critic and actor networks, respectively. The modifications from the original TD3 are $a=A_{\phi }(s)-k_{u}sign(q)+\varepsilon$ in the generation of the action and $\left(s,a,r,s^{\prime },q\right)$ in the sample buffer. q is included in the sample buffer since the actor network learns to generate the part of the control signal except for switching control.

## 4. Numerical Simulations

In this section, the performance of sliding mode RL is assessed via numerical experiments. For better assessment of the characteristics of the sliding mode RL, we considered the several different system configurations [24] which are summarized in Table 1. Each system configuration is set with different accelerations of the leader agent, disturbances, communication graphs, and delays. The considered maximum delay was 0.5 while the delay differed over each communication link. The disturbances were different for each agent, whereas the envelope of the disturbance was the same with some delay. Figure 1 shows the four communication graphs used for the simulations. Node 0 in all graphs was set as the leader node. While the graphs in the Figure 1A,B possess the same number of nodes, the graph in Figure 1B has an additional edge from node 3 to node 2. The graph in Figure 1C was constructed by adding one more node to the graph in Figure 1B. Finally, the graph in Figure 1D was included to evaluate the performance of the proposed algorithm for a relatively large communication graph. Although these graphs were considered for the effect of the graph on the proposed algorithm, the simulation results in the subsequent section indicate that the graph itself did not significantly affect the performance. The MAS was simulated at a sampling rate of 0.01 s.

The actor network consisted of a linear hidden layer with 256 nodes and an output layer with a hyper-tangent activation function. The critic network first concatenated the sub-network for the state and one for the action where the first one comprised two hidden layers with 16 and 32 nodes, and ReLU activation, and the second one consisted of one hidden layer with 32 nodes and ReLU activation. The concatenated sub-network outputs were passed to two hidden layers with 256 nodes of which output was passed to a linear output layer. The Adam optimizer was exploited to update the weight while the learning rate was 0.01 for both networks, and the batch size was 1024. The output of the actor network was clipped between −10 and 10. The update rate of the target network was set to 0.0001. The values of $\sigma ,\;\;\sigma^{\prime },\;\;c,\;\;d,$ and γ were set 2.0, 2.0, 3.0, 2, and 0.99, respectively. The state for RL was defined as $e_{x,}{}_{i}(t)$ and $e_{v,}{}_{i}(t)$ over the most recent five time steps, which resulted in a state dimension of 10. Defining the states in terms of the local error also has the advantage of enabling the use of the same network regardless of the number of neighbor agents.

The gain for switching control $k_{u}$ is an important parameter for implementing the sliding mode RL. A simulation was performed to determine proper $k_{u}$. The position and velocity of each agent were initialized using a standard normal variable. The follower agent was set to drift with disturbance without applying consensus control for a second, while the leader agent was set to move with the defined dynamics in Table 1 to realize the delay over communication links. Consensus control was then applied for 100 s, which was observed to be sufficient time for the convergence of a stable consensus algorithm. For the last 50 s, the mean squared local error (MSE) and mean squared disagreement error (MSD) were measured with 20 different initializations for each case. Table 2 shows the characteristic of the sliding mode RL with the $k_{u}$ values for cases 5 and 6. Although the performance of the sliding mode RL was assessed with $k_{u}$ values of 20 and 30, their performances were not included, since several divergences occurred for cases 5 and 6 while no divergence was observed for other cases. A larger $k_{u}$ in SMC typically increases the robustness of the control to uncertainties. However, large values of $k_{u}$ often incur excessive control at each time instance, which increases the MSE and MSD, although it is not significant. In other words, $k_{u}$ incurs a tradeoff between stability and performance at convergence. Considering that a $k_{u}$ of 40 did not incur divergence and it resulted in the smallest MSE and MSD, $k_{u}$ was set to 40.

The proposed sliding mode RL was evaluated for six cases listed in Table 1 and compared with the SMC [23], SC, modified TD3(p) [28], and slide RL [29]. The modified TD3 with a pre-trained model in [28], denoted as modified TD3(p), is TD3 with reward shaping, which uses the parameters of the trained model from a specific environment for initialization.The sliding mode RL was configured to have the same network structures and reward shaping as the modified TD3(p). Table 3 shows the MSE and MSD of the sliding mode RL and existing algorithms. The sliding mode RL provides the best MSE performance for all cases. The comparison with the modified TD3(p) on which the sliding mode RL is based clearly shows the benefit of the sliding mode, which is gained by simply structuring the control signal. Furthermore, SC outperforms SMC. It is conjectured that, while the non-switching control part may contribute to accelerating the convergence, it may introduce additional uncertainties when the state remains on the sliding surface. The SC can be considered a model-free control since the control signal is generated without explicit model knowledge. Although it required a proper definition of a sliding variable, the sliding mode RL provided better MSE than the SMC, which is based on model knowledge. This suggests that the actor network of sliding mode RL learned how to reduce the error more efficiently.

**Figure 4:**
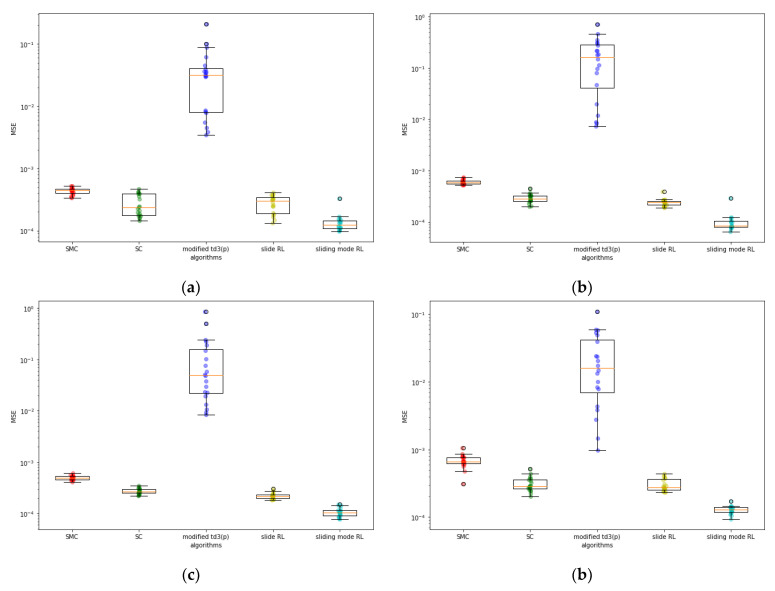
Box plot of MSEs: (**a**) case1; (**b**) case3; (**c**) case4; (**d**) case6.

**Figure 5:**
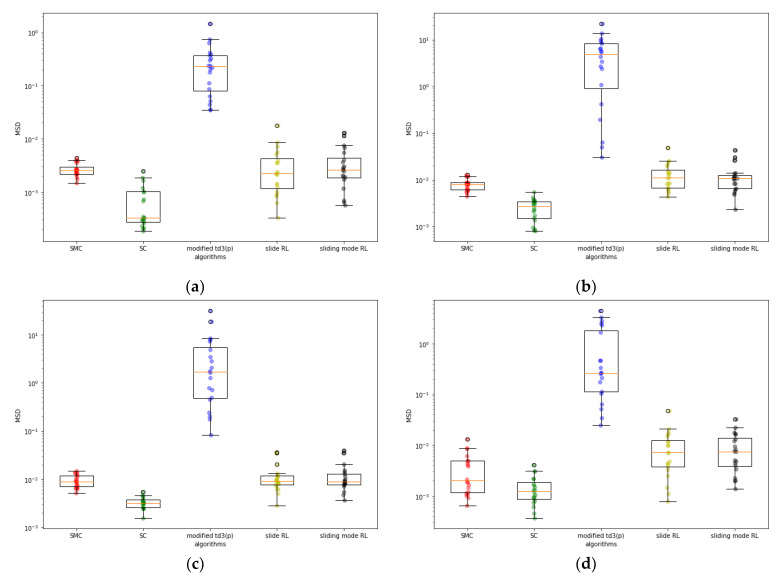
Box plot of MSDs: (**a**) case1; (**b**) case3; (**c**) case4; (**d**) case6.

While the sliding mode RL provides the best MSE performance, the SC is found to provide the best MSD performance for all cases. The sliding mode RL and the slide RL had similar MSD performance for all cases. Their MSDs were more than five times larger than those of the SC for all cases except case 4. The superior performance of SC is conjectured to be attributed to the nature of switching control without regard to any other information. Further theoretical analysis on the role of switching control remains a topic for future research. Figure 4 and Figure 5 show the box plots of the MSE and MSD, respectively. The sliding mode RL is observed to provide consistent MSE performance for various system configurations similar to the SMC, whereas the modified TD3(p) shows significant dependency on the initialization. SC and slide RL also perform consistently, although the consistency seems to depend on the system configuration. Figure 5 shows that all the consensus algorithms have a larger variance in MSD than in MSE. For the same algorithm, a small variance in MSE does not imply a small one in the MSD. Furthermore, the consistency in the MSD performance depends on the system configuration for all the algorithms considered.

## 5. Conclusions

In this study, sliding mode RL was developed by introducing a sliding variable and structured control into the modified TD3. Despite the very minor change in the modified TD3, the sliding mode RL performed significantly better than the modified TD3(p), which was the modified TD3 with pre-training. It also outperformed the SMC which used explicit model knowledge for MSE performance while it provided the best MSE among the considered consensus algorithms. It also showed that the MSD performance was comparable to that of the SMC with model knowledge.

Many interesting future research directions can be identified from the numerical experiments. The SC which may be considered as a model-free algorithm provided the best MSD performance. In addition, the sliding mode RL and slide RL significantly improve the baseline algorithm by simply introducing a sliding variable and the corresponding switching control structure. From these results, it is conjectured that one may improve the performance of RL by formulating the problem such that a proper sliding variable can be defined. In other words, the phantom of the sliding mode in RL needs to be elucidated in future research. Beyond the scope of RL, the superior performance of the SC over the SMC requires further investigation. It is conjectured that, while the non-switching control part may aid in accelerating convergence, it may work as additional uncertainties when the state remains on the sliding surface. However, a more detailed theoretical explanation may result in an opportunity to develop better nonlinear control.MAS has been proven to achieve consensus over a class of switching graphs [31]. The convergence of MAS is known to depend on the second-smallest eigenvalue of the Laplacian matrix [10]. Uncertainties are likely to have an effect on convergence speed of the consensus of MAS over graphs with time-varying connectivity. Thus, developing an RL-based consensus algorithm to accelerate the convergence of MAS over a graph with time-varying connectivity in the presence of uncertainties needs to be considered in future research.

## Figures and Tables

**Table 1:** Simulation system configurations with the acceleration of the leader agent, disturbance, communication graph, and delay. k and l are the indices of two connected nodes.

Case	Acceleration of Leader Agent	Disturbance	Graph	Delay
1	cos(7t)+cos(3t)	sin(11t)+cos(13t)	A	0.25(1+cos(t+(k+l)π/7))
2	$\left[\cos(7t)+\cos(3t)](2-e^{-t}\right)$	$\sin(11t)[3-e^{-t}]$	A	0.25(1+cos(111t+(k+l)π/7))
3	$\left[\cos(7t)+\cos(3t)](2-e^{-t}\right)$	$\sin(11t)[3-e^{-t}]$	B	$0.5{\left(1+e^{-0.1(k+l)t}\right)}^{-1}$
4	$\left[\cos(7t)+\cos(3t)](2-e^{-t}\right)$	$\sin(11t)[3-e^{-t}]$	C	$0.5(1-{\left(1+e^{-0.1(k+l)t}\right)}^{-1})$
5	$\left[\cos(7t)+\cos(3t)](2-e^{-t}\right)$	$\sin(11t)[3-e^{-t}]$	D	$0.5(1+\cos(t+(k+l)\pi /7)){\left(2+e^{-0.1(i+j)t}\right)}^{-1}$
6	$\cos(17t)(3-e^{-t}){\left(2+\cos(13t)\right)}^{-1}$	$\cos(23t)(e^{-0.t}+1)-e^{-t}$	D	$0.5(1-0.5(1+\cos(t+(k+l)\pi /7))e^{-0.1(k+l)t})$

**Table 2:** MSE and MSD with different $k_{u}$ s for cases 5 and 6.

Metrics	Case\*k_u_*	40	50	100
MSE	5	0.00011	0.00016	0.00073
	6	0.00013	0.00017	0.00088
MSD	5	0.01475	0.01521	0.01971
	6	0.00965	0.00994	0.01616

**Table 3:** Performance of the sliding mode RL for the various system configurations.

Metrics	Case\*k_u_*	1	2	3	4	5	6
MSE	SMC	0.00043	0.00041	0.00060	0.00050	0.00033	0.00069
	SC	0.00027	0.00028	0.00029	0.00027	0.00024	0.00031
	modified TD3(p)	0.04103	0.05078	0.18825	0.13413	0.02477	0.02609
	slide RL	0.00028	0.00021	0.00024	0.00022	0.00022	0.00030
	sliding mode RL	0.00014	0.00011	0.00010	0.00011	0.00011	0.00013
MSD	SMC	0.00269	0.00228	0.00802	0.00939	0.00359	0.00370
	SC	0.00069	0.00084	0.00257	0.00332	0.00217	0.00151
	modified TD3(p)	0.30884	0.36008	5.59826	4.76610	0.80414	0.99561
	slide RL	0.00361	0.00443	0.01388	0.01190	0.01442	0.01004
	sliding mode RL	0.00381	0.00397	0.01269	0.01236	0.01475	0.00965
